# Eosinophil and T cell markers predict functional decline in COPD patients

**DOI:** 10.1186/1465-9921-10-113

**Published:** 2009-11-19

**Authors:** Jeanine M D'Armiento, Steven M Scharf, Michael D Roth, John E Connett, Andrew Ghio, David Sternberg, Jonathan G Goldin, Thomas A Louis, Jenny T Mao, George T O'Connor, Joe W Ramsdell, Andrew L Ries, Neil W Schluger, Frank C Sciurba, Melissa A Skeans, Helen Voelker, Robert E Walter, Christine H Wendt, Gail G Weinmann, Robert A Wise, Robert F Foronjy

**Affiliations:** 1 Departments of Medicine and Surgery, Columbia University, New York, USA; 2 Department of Medicine, University of Maryland, Baltimore, USA; 3 Departments of Medicine and Radiology, University of California, Los Angeles, USA; 4 Departments of Medicine and Biostatistics/CCBR, University of Minnesota, Twin Cities, USA; 5 National Health and Environmental Effects Research Laboratory, Environmental Protection Agency, Research Triangle Park, USA; 6 Department of Medicine, Johns Hopkins University, Baltimore, USA; 7 Department of Medicine, Boston University, Boston, USA; 8 Department of Medicine, University of California, San Diego, San Diego, USA; 9 Department of Medicine, University of Pittsburgh, Pittsburgh, USA; 10 National Institutes of Health, Bethesda, MD, USA

## Abstract

**Background:**

The major marker utilized to monitor COPD patients is forced expiratory volume in one second (FEV1). However, asingle measurement of FEV1 cannot reliably predict subsequent decline. Recent studies indicate that T lymphocytes and eosinophils are important determinants of disease stability in COPD. We therefore measured cytokine levels in the lung lavage fluid and plasma of COPD patients in order to determine if the levels of T cell or eosinophil related cytokines were predictive of the future course of the disease.

**Methods:**

Baseline lung lavage and plasma samples were collected from COPD subjects with moderately severe airway obstruction and emphysematous changes on chest CT. The study participants were former smokers who had not had a disease exacerbation within the past six months or used steroids within the past two months. Those subjects who demonstrated stable disease over the following six months (ΔFEV1 % predicted = 4.7 ± 7.2; N = 34) were retrospectively compared with study participants who experienced a rapid decline in lung function (ΔFEV1 % predicted = -16.0 ± 6.0; N = 16) during the same time period and with normal controls (N = 11). Plasma and lung lavage cytokines were measured from clinical samples using the Luminex multiplex kit which enabled the simultaneous measurement of several T cell and eosinophil related cytokines.

**Results and Discussion:**

Stable COPD participants had significantly higher plasma IL-2 levels compared to participants with rapidly progressive COPD (p = 0.04). In contrast, plasma eotaxin-1 levels were significantly lower in stable COPD subjects compared to normal controls (p < 0.03). In addition, lung lavage eotaxin-1 levels were significantly higher in rapidly progressive COPD participants compared to both normal controls (p < 0.02) and stable COPD participants (p < 0.05).

**Conclusion:**

These findings indicate that IL-2 and eotaxin-1 levels may be important markers of disease stability in advanced emphysema patients. Prospective studies will need to confirm whether measuring IL-2 or eotaxin-1 can identify patients at risk for rapid disease progression.

## Background

Research has indicated that eosinophils[1] and T lymphocytes[2,3] are important determinants of disease stability in COPD patients. Given these studies, we sought to determine if eosinophil or T cell related cytokine levels measured from the lung lavage and plasma of advanced COPD patients could predict the future clinical course of their disease. Our analyses in this study were primarily focused on the role of IL-2, IL-2R, RANTES and Eotaxin-1 as these cytokines are critical regulators of T cell and eosinophil proliferation and migration[4,5]. Currently, there are no tests that can reliably identify which patients are more likely to deteriorate over time. Forced expiratory volume in one second (FEV1) is used to diagnose the stage of chronic obstructive pulmonary disease (COPD) and to predict COPD mortality [6,7]. However, FEV1 is a physiologic parameter that changes relatively slowly over time in COPD patients[8] and a given value of FEV1 does not accurately predict the short or long-term course of a patient's disease. The discovery of new markers that would correlate with disease severity and foretell progression would not only enable clinicians to identify susceptible patients but would also allow researchers, by monitoring marker levels, to more readily identify therapies that may have a beneficial effect on the outcome of this disease.

In this study, we retrospectively analyzed cytokine levels in the lung lavage and plasma of participants that were enrolled in the NIH-sponsored FORTE trial (Feasibility of Retinoids for the Treatment of Emphysema). The study participants were stable but advanced emphysema patients who had not smoked or had a respiratory exacerbation for at least six months prior to study entry. At baseline and before study drug treatment, lung lavage and plasma samples were obtained from the study participants who subsequently underwent extensive lung testing over a nine-month time period. To determine if eosinophil or T cell cytokine levels were associated with the rate of decline of lung function, we analyzed a subset of participants who experienced a significant decline in lung function (>10% decrease in % predicted FEV1 post-bronchodilator; n = 16) during the first six months of the study. The results obtained from this group were compared with study participants with stable disease (no decrease in % predicted FEV1 post-bronchodilator; n = 34), age-matched controls (plasma samples; n = 11) and non-age matched controls (lung lavage; n = 8).

## Materials and methods

### Selection Criteria for Study Participants

**Figure 1:**
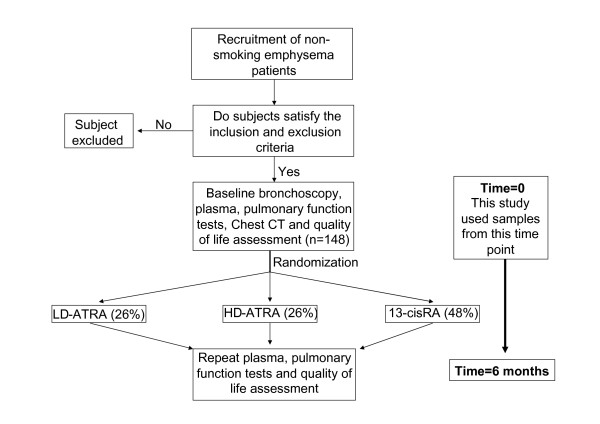
**Outline of Study Methodology**.

**Figure 2:**
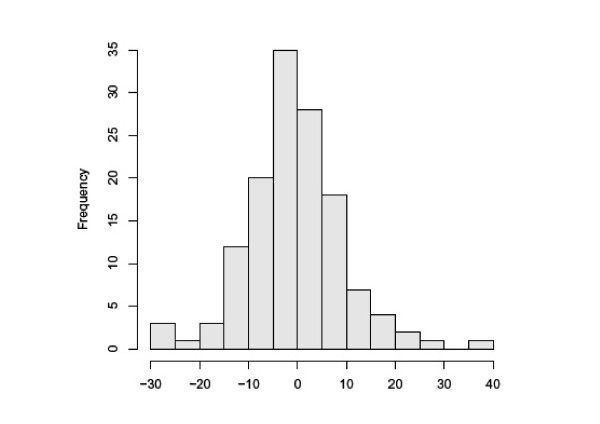
**Distribution of Δ % Predicted FEV1 at the 6 Month Time Point**. The bar graph represents the frequency of distribution of Δ % predicted FEV1 at the six month time point. Most participants (approximately 63%) demonstrated stable disease with the % predicted FEV1 varying less than 5% from baseline. Less than 20% of participants had an absolute decline in % predicted FEV1 of 10% or greater.

Emphysema subjects were FORTE study participants [9]. Entry criteria included age > 45 years, FEV_1 _25 to 80% of predicted, diffusing capacity of the lung for carbon monoxide (DLco) ≤ 80% of predicted, visual evidence of emphysema occupying ≥ 10% of the lung on CT scan, and willingness to undergo bronchoscopy. Participants were excluded for a Karnofsky score < 70%; excessive airway hyperreactivity; resting oxygen saturation < 90% or Pco_2 _> 45 mm Hg; use of systemic corticosteroids within 2 months or tobacco within 6 months; hyperlipidemia; a history of clinical depression; concurrent use of medications that alter the metabolism of retinoids; or other significant illnesses including cancer, liver disease, or heart failure. Women of child-bearing potential were required to use two forms of contraception or abstinence. After enrollment, baseline bronchoscopy, blood tests, Chest CT, pulmonary function tests and quality of life assessments were performed and then participants were randomized to low dose all trans-retinoic acid (LD-ATRA; 1 mg/kg), high dose ATRA (HD-ATRA; 2 mg/kg), 13-cis retinoic acid (13-cRA; 1 mg/kg) or placebo for six months (Figure 1). This study utilized the baseline plasma analyses that were obtained prior to study drug administration. Importantly, drug treatment had no effect on ΔFEV1, CT density score or health related quality of life in this study[9]. Figure 2 demonstrates the distribution of rate of decline of % predicted FEV1 over the first six months of the study. Of the 148 study participants, nineteen experienced an absolute decline of at least 10% in their predicted FEV1 over the first six months of the trial. Of these nineteen participants, 16 had stored plasma samples available for further analyses with the Luminex system (ΔFEV1 % predicted = -16.0 ± 6.0). Since this study aimed to compare eosinophil and T cell cytokine patterns between subjects with progressive disease vs. stable COPD subjects, we compared this group to a subset of FORTE subjects who demonstrated disease stability during this same time period (Δ % predicted FEV1 = 4.7 ± 7.2). Likewise, lavage samples from the rapid decliners (n = 8 for lung lavage) were compared with lavage samples from eleven randomly selected study participants with no decline in % predicted FEV1 over the first six months. Normal controls values for plasma (n = 11) and lung lavage (n = 8) were obtained from non-smoking volunteers that had no significant respiratory disease. Of note, at the nine month follow up time point, the rapid decliners continued to demonstrate a decreased % predicted FEV1 (-7.8 ± 4.8) compared to the stable COPD participants (2.3 ± 5.1). Demographic data on all the study participants is provided in Table 1, Table 2, Table 3 and Table 4. Written consent was obtained from all study participants and the institutional review boards of all of the participating centers approved the trial.

**Table 1:** Demographics of Entire Cohort of FORTE Study Participants

	FORTE Subjects	
	
	Mean	Std
**N**	148	

**Age at Randomization, years**	65.8	7.4

**Gender, % male**	58.1	

**Smoking HX, pack-years**	57.8	29

**BL Chronic cough, % subjects**	24.5	

**BL StGeo Total Score**	39.3	13.1

**BL Post-BD %Pred FEV1**	42.5	13.7

**BL Post-BD %Pred FVC**	80.1	15.7

**Bronchodilator response, % changed**	12.7	10.4

**BL %Pred TLC**	118.1	16.2

**BL %Pred RV (meth A)**	180.8	48.1

**BL %Pred DLCO**	37.1	12.0

**DLCO/VA, %Pred**	46.3	16.1

**BL CT Score, %emph**	38.5	12.8

Data is expressed as mean ± standard deviation

Demographics of the entire cohort of 148 FORTE Study Participants.

**Table 2:** Demographics of Stable and Rapidly Progressive COPD Subjects.

	Stable	Rapid Decliners
Age	65.0	65.8

Male gender percentage	56.67 (17 out of 30)	62.5 (10 out of 16)

Cigarette pack-years	56.5	46.6

SGRQ total score	32.76	29.96

Baseline pulmonary function		

FEV1% predicted	41.33	43.57

FVC % predicted	74.43	81.47

Bronchodilator response % change	10.59	9.56

DLCO % predicted	38.0	35.4

**Table 3:** Demographics of Normal Controls for Plasma.

	Normal Controls for Lavage
N	8

Age	24.6 ± 4.0

Sex	62.5% Male

White	87.5%

African American	12.5%

Age is represented as mean ± standard error of measurement

**Table 4:** Demographics of Normal Controls for Lavage.

	Normal Controls for Plasma
N	11

Age	53.8 ± 13.5

Sex	63.6% Male

Smoking history	36.4%

White	63.6%

African American	18.2%

Hispanic	18.2%

Age is represented as mean ± standard error of measurement

### Bronchoscope Procedure

Fiberoptic bronchoscopy was performed on an outpatient basis in the endoscopy units of the participating centers of this trial as per standard protocol. All participants, received albuterol 2.5 mg and atrovent 1.0 mg by hand held nebulizer prior to their bronchoscopy. During the procedure, participants had continuous monitoring of pulse oximetry, vital signs and received oxygen via nasal cannula as required. Local anesthesia was provided by administering viscous lidocaine to the nasopharynx and 2% lidocaine instilled via the bronchoscope to the vocal cords and tracheobronchial tree. Participants were sedated by use of 2-5 mg of midazolam IV at the discretion of the bronchoscopist. The bronchoscope was inserted nasally when possible, and the oral route was used as a second choice. BAL was performed by instilling 180-240 ml of saline solution into the medial or lateral segment of the right middle lobe, with a dwell time of up to 30 seconds, followed by aspiration. A target goal was to obtain a return of at least 60 ml of lavage fluid. Following bronchoscopy, participants were observed with regular monitoring of oximetry and vital signs. Participants were discharged after a minimum of 2 hours of observation, once safe swallowing had returned and observations were satisfactory. All were given an emergency contact number and followed up within 2 weeks. Severe adverse events were documented at the time of bronchoscopy and reported promptly to the data safety monitoring board for the trial.

### Processing of Lung Lavage and Plasma Samples

The lung lavage fluid was filtered through a sterile 100-micron nylon mesh (Falcon) to remove mucus and debris. The fluid was then centrifuged at 200 × *g *for 15 minutes at 4°C. The cellular pellet was processed for RNA extraction and the lavage supernatant was aliquoted and immediately frozen at -70°C to -80°C and stored on-site. When ready for analysis, aliquots were shipped frozen to testing sites for biomarker determination. Baseline plasma samples were obtained from the study participants. Approximately 30 ml of blood was obtained via venipuncture into three 10 ml heparinized tubes. These tubes were then centrifuged at 200 × *g *for 8 minutes at 4°C. The plasma was transferred into labeled 1.5 ml tubes and stored at -70°C to -80°C and stored on-site until they were ready to be shipped as described above.

### Pulmonary Function Testing

Pre- and post-bronchodilator pulmonary function testing (PFT) was performed at the screening visit, at baseline, 3 month, 6 month and 9 month visits on all patients. Spirometry was performed pre- and post-bronchodilator at each visit while diffusing capacity (DLCO) was performed post BD at each visit. Pre-BD testing was done at least four hours after the use of short acting bronchodilators (albuterol, fenoterol) and at least 12 hours after the use of long-acting bronchodilators (theophylline or salmeterol). Post-BD testing took place at least 15 minutes and no longer than 1 hour after 2 inhalations of albuterol. Testing was completed within sixty minutes of bronchodilator administration. Bronchodilators were administered via a metered dose inhaler under the supervision of a trained pulmonary function technologist. Spirometry was performed in adherence to ATS recommendations[10,11]. Predicted values for FEV1 were based on the prediction equations of Hankinson et al[11]. Single breath diffusing capacity (DLCO) was performed following standard techniques[12]. Normal reference values were derived from those of Crapo and colleagues[13]. The mean of three acceptable maneuvers is reported as the data point.

### Cytokine Measurements

Plasma and lung lavage cytokines were measured using the Luminex human cytokine multiplex-25 bead array assay kit (Biosource, Camarillo, CA). This kit is able to simultaneously measure human IL-1β, IL-1Ra, IL-2, IL-2R, IL-4, IL-5, IL-6, IL-7, IL-8, IL-10, IL-12p40/p70, IL-13, IL-15, IL-17, TNF-α, IFN-α, IFN-γ, GM-CSF, MIP-1α, MIP-1β, IP-10, MIG, Eotaxin-1, RANTES, and MCP-1. The 25 multiplex array was chosen since it would measure several Th1/Th2 and eosinophil related cytokines. Standard curves for each cytokine were generated by using the reference cytokine concentrations supplied in this kit. Incubation buffer (50 μL) and 1:2 diluted plasma or lung lavage fluid samples or standards (50 μL) were pipetted into the wells and incubated for 2 hours with the beads. All samples and standards were performed in duplicate. The wells were then washed using a vacuum manifold and biotinylated detector antibody was subsequently added. After 1 hour, the beads were washed again and then incubated for 30 minutes with streptavidin conjugated to the fluorescent protein, R-phycoerythrin (Streptavidin-RPE). After washing to remove the unbound Streptavidin-RPE, the beads (minimum of 50 beads per cytokine) were analyzed using a Luminex 100 instrument (Upstate, Temecula, CA), which monitored the spectral properties of the beads while simultaneously measuring the amount of fluorescence associated with R-phycoerythrin. Raw data (mean fluorescence intensity, MFI) were analyzed using MasterPlex software (Upstate, Temecula, CA). Luminex analyses focused specifically on plasma IL-2 and eotaxin-1 were conducted on an additional twenty-three COPD subjects and eight normal controls. These controls were repeat samples from our first analyses that were utilized to demonstrate reproducibility of our results. All luminex analyses were conducted by Ocean Ridge Biosciences (ORB, Jupiter, Florida).

### Statistical Analysis

The results are presented as the mean ± standard error for all variables that were examined. Analyses demonstrated that variances were equal for measurements of IL-2 and eotaxin-1. Comparisons between groups were done using ANOVA for non-repeated measures and significance and the null hypothesis was tested at the 5% level.

## Results

### Plasma Cytokine Levels in COPD Participants and Normal Controls

In our initial analyses, we examined twenty-five plasma cytokine levels (IL-1β, IL-1Ra, IL-2, IL-2R, IL-4, -5, -6, -7, -8, -10, -12p40/p70, -13, -15, -17, TNF-α, IFN-α, IFN-γ, GM-CSF, MIP-1α, MIP-1β, IP-10, MIG, Eotaxin-1, RANTES, MCP-1) and found that nineteen of these were elevated in the COPD participants (n = 11) relative to age-matched normal controls (n = 11) (Table 5). However, this elevation was statistically significant (p < 0.05) when compared to normals for only nine of these cytokines (IL-4, -5, -7, -8, IFN-α, GM-CSF, MIP-1α, MIP-1β and IP-10). IL-10 was the only cytokine that trended lower in the COPD groups although this again did not reach statistical significance.

**Table 5:** Comparisons between Plasma Cytokine Levels in COPD.

	NORMALS (N = 11)	EMPHYSEMA (N = 27)	p value
IL-1β	151(55)	329(58)	NS

IL-1Ra	1665(589)	2933(575)	NS

IL-2*	27(11)	49(8)	NS

IL-2R*	511(162)	631(95)	NS

IL-4	**21(9)**	**62(11)**	**<0.04**

IL-5	**9(4)**	**26(5)**	**<0.05**

IL-6	47(17)	74(10)	NS

IL-7	**37(16)**	**97(13)**	**<0.02**

IL-8	**10(2)**	**18(2)**	**<0.04**

IL-10	78(52)	47(10)	NS

IL-12p40/p70	503(53)	590(60)	NS

IL-13	17(9)	33(9)	NS

IL-15	101(48)	171(26)	NS

IL-17	46(24)	107(20)	NS

TNF-α	69(22)	75(15)	NS

IFN-α	**70(41)**	**264(43)**	**<0.02**

IFN-γ	73(32)	158(26)	NS

GM-CSF	**192(71)**	**423(59)**	**<0.04**

MIP-1α	**119(19)**	**192(20)**	**<0.05**

MIP-1β	**831(235)**	**1771(230)**	**<0.03**

IP-10	**60(12)**	**95(8)**	**<0.04**

MIG	505(247)	819(120)	NS

EOTAXIN*	1043(237)	659(77)	=0.06

RANTES	30969(4420)	33527(3411)	NS

MCP-1	1931(158)	1776(98)	NS

Plasma levels of 25 human cytokines were measured in COPD participants (n = 27) and age-matched normal controls (n = 11) using the Luminex 25-plex assay. Additional analyses for plasma IL-2, IL-2R and Eotaxin-1 were conducted on 6 rapid decliners and 17 stable COPD subjects. Significant changes in cytokine levels were found in nine of the examined cytokines (indicated in bold, p < 0.05). Parentheses indicate standard error of measurement.

*N = 11 for normal controls and N = 50 for emphysema subjects

Data is reported as mean ± standard error of measurement

The standard error of measurement is in parentheses

### Plasma Cytokine Levels in Stable or Progressive COPD

**Figure 3:**
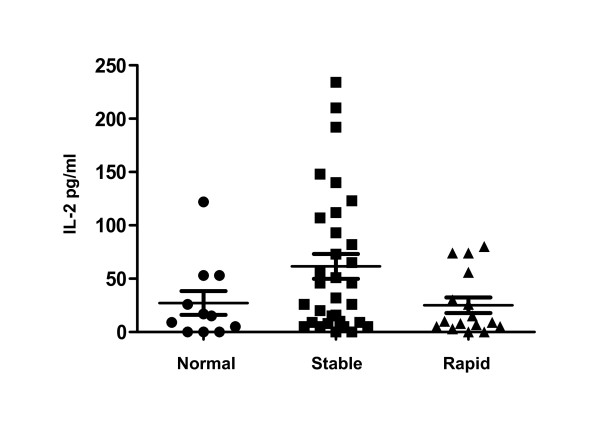
**IL-2 Levels are Increased in Stable COPD Participants**. Plasma levels of IL-2 were significantly increased in stable COPD participants (black squares, n = 34) compared to subjects with rapidly progressive COPD (black triangles, n = 1) (p = 0.04) and trended higher in stable COPD subjects compared to age-matched normal controls (black circles, n = 11) (p = 0.11).

**Figure 4:**
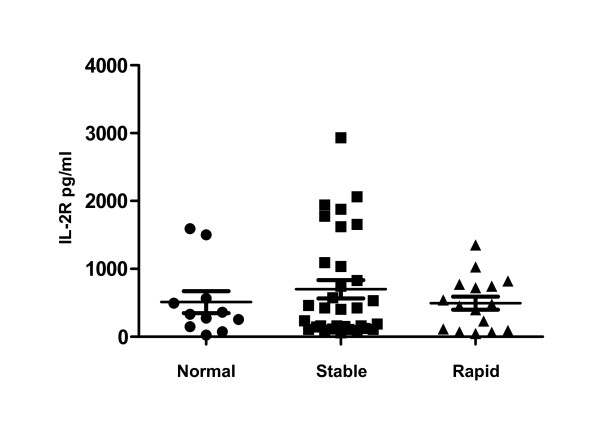
**IL-2R Levels in Stable and Rapidly Progressive Cohorts**. Plasma levels of IL-2R were not significantly altered in any of the groups we examined though the highest IL-2R levels were measured from subjects with stable COPD (black squares).

**Figure 5:**
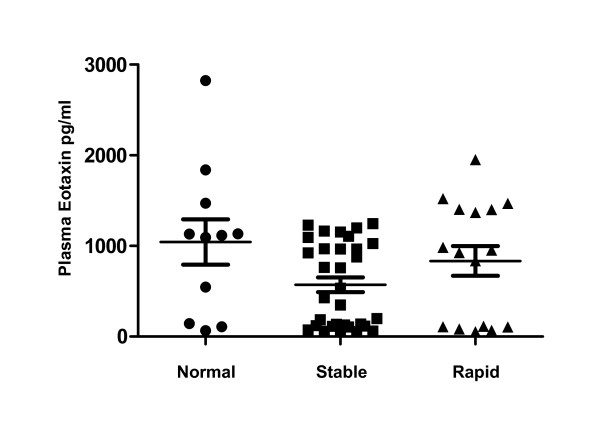
**Eotaxin-1 Levels are Decreased in Subjects with Stable COPD**. Plasma levels of eotaxin-1 were significantly lower in stable COPD participants (black squares, n = 34) compared to age-matched normal controls (black circles, n = 11) (p < 0.04). In addition, subjects with rapidly progressive COPD (black triangles, n = 16) tended to have higher levels compared to those with stable disease though this difference did not reach statistical significance (p = 0.11).

Initial multiplex analyses revealed that cytokine levels were increased in individuals with stable COPD compared to those with rapidly progressive COPD (Table 6). These initial studies found that plasma IL-2 was significantly increased in stable COPD subjects compared to those with rapidly progressive disease while plasma eotaxin-1 levels were significantly lower in stable COPD subjects compared to controls. Confirmatory studies specifically examining plasma IL-2, IL-2R and eotaxin-1 were conducted on an additional 17 stable and 6 rapidly progressive COPD subjects. Individuals with stable COPD had IL-2 plasma levels (Figure 3) that were nearly three-fold increased compared to those with rapidly progressive COPD (p = 0.04) and normal controls (p = 0.11). The levels of IL-2 in the rapidly progressive COPD group were comparable to the levels seen in the normal controls. In contrast, there were no significant differences in IL-2R levels between any of the study groups (Figure 4). However, every COPD subject with a plasma IL-2 >100 pg/ml or IL-2R >1500 pg/ml demonstrated a stable disease course. Eotaxin-1 levels, on the other hand, were significantly lower in the stable COPD group (Figure 5) compared to normal controls (p < 0.03) and trended lower in stable COPD subjects compared to those with rapidly progressive disease (p = 0.11). Indeed, a plasma eotaxin-1 of >1300 pg/ml was predictive of a more rapid disease progression.

**Table 6:** Comparison of Plasma Cytokine Levels between Rapid Decliners Stable COPD Participants and Normal Controls.

	NORMALS (N = 11)	STABLE (N = 17)	DECLINERS (N = 10)	p value Stable vs. Rapid	p value Stable vs. Normals
IL-1β	151(55)	414(104)	184(61)	<0.06	**<0.03**

IL-1Ra	1665(589)	3514(1078)	1945(598)	NS	NS

IL-2*	28(12)	**62(11)**	**25(7)**	**<0.04**	=0.10

IL-2R*	511(162)	700(128)	495(97)	NS	NS

IL-4	**21(9)**	**70(22)**	48(11)	NS	**<0.04**

IL-5	**9(4)**	**32(9)**	16(4)	NS	**<0.03**

IL-6	47(17)	85(20)	56(10)	NS	NS

IL-7	37(16)	101(25)	97(13)	NS	**<0.03**

IL-8	10(2)	20(4)	15(2)	NS	**<0.03**

IL-10	78(52)	51(17)	40(16)	NS	NS

IL-12p40/p70	503(53)	592(117)	586(58)	NS	NS

IL-13	17(9)	42(16)	19(8)	NS	NS

IL-15	101(48)	203(51)	117(20)	NS	NS

IL-17	46(24)	123(38)	80(18)	NS	NS

TNF-α	69(22)	88(28)	53(12)	NS	NS

IFN-α	**70(41)**	**308(83)**	188(48)	NS	**<0.02**

IFN-γ	**73(32)**	**187(50)**	109(22)	NS	**<0.05**

GM-CSF	**192(71)**	**463(120)**	356(63)	NS	**<0.05**

MIP-1α	**119(19)**	**206(36)**	168(30)	NS	**<0.04**

MIP-1β	**831(235)**	**1976(431)**	1422(243)	NS	**<0.03**

IP-10	**60(12)**	**88(11)**	106(18)	NS	**<0.05**

MIG	505(247)	941(235)	610(87)	NS	NS

EOTAXIN*	**1043(237)**	**572(128)**	834(164)	=0.11	**<0.04**

RANTES	30969(4420)	30066(4135)	39411(7420)	NS	NS

MCP-1	1931(158)	1771(181)	1785(129)	NS	NS

*N = 34 stable COPD subjects, 16 rapid COPD subjects and 11 normal controls

Data is reported as mean ± standard error of measurement

The standard error of measurement is in parentheses

Plasma cytokine levels were measured as above in stable COPD participants (n = 17) and COPD participants who demonstrated rapid decline in the first six months of the trial (n = 10). For IL-2, IL-2R and Eotaxin-1 plasma levels were determined in an additional 6 rapid decliner and 17 stable COPD subjects. IL-2 was the one cytokine that was significantly increased in stable COPD subjects compared to rapid decliners (indicated in bold, p < 0.05). Parentheses indicate standard error of measurement.

### Lung Lavage Cytokine Levels in COPD Patients and Controls

**Figure 6:**
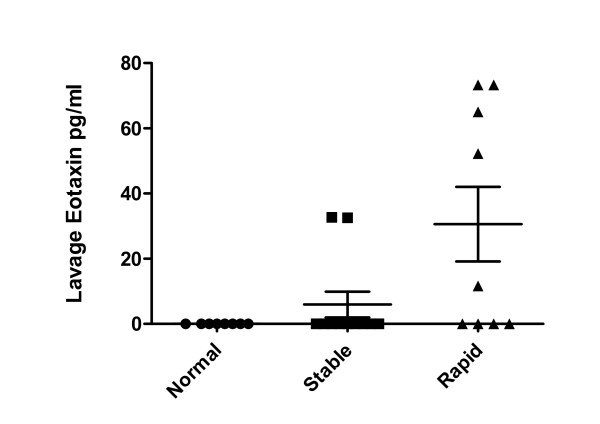
**Lung Lavage Eotaxin-1 Levels in COPD Subjects, Asthmatics and Normal Controls**. Lung lavage Eotaxin-1 levels measured using the Luminex 25-plex assay were significantly higher in rapidly progressive COPD participants (black triangles, n = 9) compared to normal controls (black circles, n = 8) (p < 0.03). In addition, levels in participants with rapidly progressive COPD had higher levels than participants with stable COPD (black squares, n = 11) (p < 0.05).

**Figure 7:**
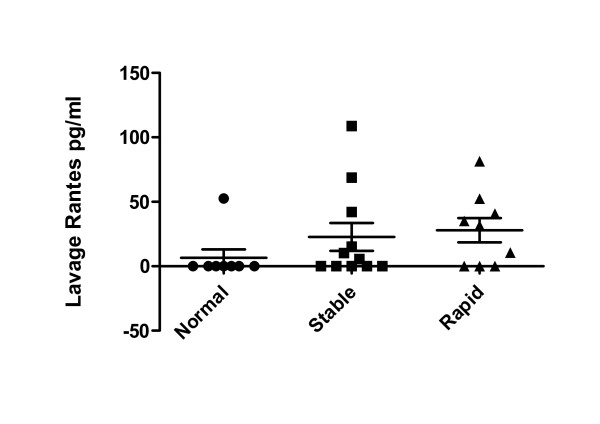
**Lung Lavage RANTES Levels in COPD Subjects, Asthmatics and Normal Controls**. Lung lavage RANTES levels were measured in stable COPD participants (black squares, n = 11), rapidly progressive COPD participants (black triangles, n = 9) and normal controls (black circles, n = 8) using the Luminex 25-plex assay. Increases were seen in both cohorts of COPD; however, these differences did not reach statistical significance.

Of the twenty-five cytokines tested only eight (IL-1Ra, IL-2, -6, -8, IP-10, RANTES, MCP-1 and eotaxin-1) had detectable levels within the lung lavage. Eotaxin-1, however, was the only cytokine that differed significantly amongst the groups tested (see Table 7). Eotaxin-1 levels (Figure 6) were significantly higher in the rapidly progressive cohort compared to the stable COPD group (p = 0.04) and to normal controls (p < 0.02). In addition, the COPD participants as a group had significantly higher levels of eotaxin-1 than normal controls (p < 0.01). Of note, every COPD subject with a lavage eotaxin-1 level >50 pg/ml demonstrated rapid disease progression. Elevations in RANTES levels (Figure 7) were noted in both the stable and rapid COPD groups; however, these differences were not statistically significant.

**Table 7:** Lung Lavage Cytokine Levels

	IL-1Ra	IL-2	IL-6	IL-8	IP-10	RANTES	MCP-1	Eotaxin
Rapid COPD	30.1(36.7)	27.4(43.2)	31.7(38.0)	32.7(23.9)	24.8(17.7)	28.0(28.1)	25.3(37.6)	**30.6(34.3)**

Stable COPD	9.0(22.3)	13.6(37.0)	20.6(38.2)	18.0(25.4)	15.8(17.6)	22.6(38.0)	8.3(23.4)	**5.9(13.2)**

Normal Controls	16.4(39.0)	27.5(51.3)	16.6(47.0)	38.2(36.3)	39.3(29.3)	6.6(18.6)	9.3(26.2)	**0(0)**

n = 9 for rapid decliners, n = 11 for stable COPD subjects, n = 8 for normal controls

Data is reported as mean ± standard error of measurement

The standard error of measurement is in parentheses

Lung lavage levels of 25 human cytokines were measured in stable COPD participants (n = 11), rapidly progressive COPD participants (n = 9) and normal controls (n = 8) using the Luminex 25-plex assay. Eight of these cytokines had detectable levels within our samples. Significant changes in cytokine levels between stable and rapid decliners were detected for eotaxin-1 (indicated in bold, p < 0.05). Parentheses indicate standard error of measurement.

## Discussion

This study demonstrates that markers of T cell and eosinophilic inflammation are predictive of disease progression of COPD. Individuals with stable disease have higher plasma levels of IL-2 than those with rapidly progressive COPD and lower plasma eotaxin-1 levels compared to normal controls. In addition, those COPD subjects who experienced a subsequent physiologic deterioration of their disease had markedly higher lung lavage eotaxin-1 levels compared to subjects who demonstrated disease stability over the same time interval. Together, these results suggest that measuring IL-2 and eotaxin-1 levels could enable physicians to identify those COPD patients that require more intensive monitoring and treatment in the future. Moreover, these findings indicate that cell-mediated immune responses have an important effect on the clinical status of this disease.

IL-2 is a Th1 derived cytokine that induces the proliferation and activation of both CD4+ and CD8+ lymphocytes. While several recent studies, have implicated T lymphocytes in the pathogenesis[3,14] and functional decline[15,16] of COPD, the exact role they play in this disease remains ambiguous. In fact, activation of peripheral CD4+ cells correlates positively with lung function in smokers[17]. Moreover, smokers with preserved lung function have a prominent up-regulation of T regulatory cells in the lung compared to never smokers and patients with COPD[18]. In this study we found that the Th1 cytokine IL-2 was significantly elevated in the plasma of COPD patients who demonstrated disease stability over a six-month time period. Together, these data suggest that T cell mediated immune responses can alter the physiologic progression of this disease.

IL-2 may prevent disease progression by promoting virus-specific CD4+ and CD8+ T-cell responses which deter virus replication and thereby limit the damaging effects of chronic viral infection in the lung[19]. CD8+ cells are increased in the lungs of guinea pigs with latent adenoviral infection[20] and this increase may act to reduce lung inflammation by suppressing active viral infection[21]. Respiratory syncytial virus (RSV) diminishes the effector activity of CD8+ cells and the development of CD8+ T cell memory[22]. This effect, however, can be reversed by IL-2[23] thus preventing recurrent infection with this common pathogen in patients with COPD[24,25]. In addition to viruses, cytotoxic lymphocyte responses, which are coordinated by CD4+ cells, exert an important role in defending against H. influenza infections in the lung[26]. In fact, studies in mice demonstrate that cigarette smoke alters T cell function which can render the animal more susceptible to infection [27]. Thus, we postulate that enhanced T cell responses in our stable COPD cohort may have acted to prevent disease progression by limiting the pathogenicity of bacterial and viral infections within the lung.

Another means by which IL-2 may influence disease progression is by regulating the survival of T cells[28]. In culture, IL-2 promotes T cell survival in part by inducing the expression of Bcl-2, a protein that protects from passive apoptotic cell death (PCD)[29,30]. T lymphocyte apoptosis is increased both in the peripheral blood[31] and lung lavage[32] of COPD patients. The loss of these T cells can render the lung susceptible to infections[33,34] thereby increasing the likelihood of disease exacerbations, an important factor in the progression of the disease[35]. In addition, the uncleared apoptotic cells can injure the lung by releasing proteases and other harmful intracellular contents[36]. These damaging effects are accentuated by the fact that pulmonary macrophages from COPD patients have a defect in their ability to phagocytose apoptotic cells in the lung[37]. Conversely, it is conceivable that IL-2 protects the lung by actually stimulating the apoptosis of auto-reactive T lymphocytes. IL-2 has been shown to program mouse lymphocytes for apoptosis and mice deficient in IL-2Rα are resistant to Fas-mediated activation induced cell death (AICD)[38]. Activation induced cell death is a critical process for maintaining self-tolerance[39]. IL-2 by activating AICD can eliminate autoreactive T cells and prevent the development of inflammatory responses to self antigens which are capable of generating emphysematous changes in the lung[40].

In contrast to IL-2, increases in eotaxin-1 were associated with disease progression in COPD. We found significant increases in lung lavage eotaxin-1 levels in COPD patients compared to normal controls. More importantly, those patients whose lung function subsequently declined over the ensuing six months had significantly higher lavage eotaxin-1 levels than those subjects with stable lung function over the same time period. In addition, disease stability was associated with decreased plasma eotaxin-1 levels. Eotaxin-1 is a CC chemokine (CCL11) that binds to the CC chemokine receptor 3 (CCR3) on the surface of eosinophils thereby inducing eosinophil activation[41] and migration[42]. Lung eosinophilia has been linked with bronchial hyperreactivity in COPD patients[1]. Moreover, the expression of both eotaxin-1 and CCR3 is up regulated during exacerbations of chronic bronchitis[43] and eotaxin-1 levels are associated with bronchodilator response and the extent of emphysema on CT scans[44]. Coupled with these previous findings, our data indicate that eotaxin-1-mediated lung eosinophilia may be a critical factor in the progression of this disease.

It is important to note that all the study participants at baseline were former smokers who were clinically stable and had no signs of exacerbation or recent infection. In fact, the presence of an exacerbation was an exclusion criterion for the trial. Thus, we cannot ascribe the subsequent decline in FEV1 in the rapid decliners to the presence of disease exacerbation or inherent differences with the stable COPD cohort. Indeed, both the rapid decliners and stable COPD subjects selected for these studies had GOLD IIB disease with visual evidence of emphysema occupying ≤ 10% of the lung on CT scan. The subjects did not use steroids for at least two months prior to study entry and did not have excessive airway hyperreactivity during bronchodilator testing. Similarly, our study findings cannot be attributed to the study drug-retinoic acid. Plasma and lavage measurements were taken at baseline prior to initiation of retinoic acid and retinoic acid itself had no impact on any of the physiologic, radiographic or quality of life measures at the six or nine-month time point[9].

Given the multiple analyses that were conducted it is conceivable that the changes in IL-2 may have occurred by chance. However, further plasma IL-2 analyses on an additional 6 rapid decliners and 17 stable COPD subjects confirmed the differences between these two groups. However, prospective analyses will be needed to validate these results and determine if these findings can be extrapolated to a more heterogeneous population of COPD subjects. A strength of this study is that it contains both plasma and lung lavage analyses on a well-characterized cohort of previously stable advanced emphysema subjects. The literature regarding the impact of T cell and eosinophil related cytokines in advanced emphysema is limited-particularly for lung lavage. In fact, this is one of the only studies to examine the relationship between a lung lavage biomarker and subsequent rate of decline of lung function in COPD[45]. Thus, our findings provide important novel evidence that these cell types are involved in the progression of the disease.

## Conclusion

In summary, in this study we have identified distinct differences in cytokines levels in advanced emphysema patients whose disease progressed rapidly over a six-month time period. The changes in IL-2 and eotaxin-1 suggest that alterations in T lymphocyte and eosinophil trafficking in the lung could be important factors affecting the stability of this disease. If confirmed in a larger prospective trial, these results could lead to the development of useful clinical biomarkers that could accurately predict the future course of the disease. This would not only permit clinicians to ascertain which patients require closer observation but would also provide researchers with a surrogate endpoint to detect clinically important responses to therapies.

## List of Abbreviations

CT: Computed Tomography; BAL: bronchoscopic alveolar lavage; FEV1: Forced expiratory volume in one second; COPD: chronic obstructive pulmonary disease; MMPs: matrix metalloproteinases; PFT's: pulmonary function tests; HRCT: high resolution computed tomography; RANTES: Regulated upon activation, normal T cell expressed and secreted; AICD: Activation Induced Cell Death; PCD: Passive Cell Death.

## Competing interests

The authors declare that they have no competing interests.

## Authors' contributions

JD contributed to the study design and the acquisition and interpretation of data.

SS contributed significantly to the study design and execution and aided in the preparation of the manuscript and the statistical analysis of the data.

MR contributed to the study design and analysis of data.

JC helped to design the study and analyze the data. AG was instrumental in collecting clinical samples for the study.

DS was instrumental in collecting clinical samples for the study.

JG contributed significantly to the study design and execution.

TL contributed significantly to the study design and execution.

JM contributed significantly to the study design and execution.

GO contributed significantly to the study design and execution and preparation of the manuscript.

HV contributed to the statistical analysis of the data.

JR contributed significantly to the study design and execution and aided in the preparation of the manuscript.

AR contributed significantly to the study design and execution and aided in the preparation of the manuscript.

NS contributed significantly to the study design and execution. FS contributed significantly to the study design and execution. MS contributed significantly to the study design and execution. RW contributed significantly to the study design and execution. CW contributed significantly to the study design and execution. GW contributed significantly to the study design and execution. RAW contributed significantly to the study design and execution and aided in the preparation of the manuscript.

RF contributed significantly to the study design and execution. In addition, he prepared the manuscript and the statistical analysis of the data.
